# Preparation of topical bimatoprost with enhanced skin infiltration and *in vivo* hair regrowth efficacy in androgenic alopecia

**DOI:** 10.1080/10717544.2022.2027046

**Published:** 2022-01-18

**Authors:** Laxman Subedi, Prashant Pandey, Jung-Hyun Shim, Ki-Taek Kim, Seung-Sik Cho, Kyo-Tan Koo, Beum Joon Kim, Jin Woo Park

**Affiliations:** a Department of Biomedicine, Health & Life Convergence Sciences, BK21 Four, Biomedical and Healthcare Research Institute, Mokpo National University, Jeonnam, Republic of Korea; b College of Pharmacy and Natural Medicine Research Institute, Mokpo National University, Jeonnam, Republic of Korea; c BioBelief Co., Ltd., Seoul, Republic of Korea; d Department of Dermatology, Chung-Ang University College of Medicine, Seoul, Republic of Korea

**Keywords:** Bimatoprost, prostaglandins, supersaturated topical delivery system, highly skin-permeable, alopecia, hair growth, hair follicles

## Abstract

To prepare a topical formulation of bimatoprost (BIM) with high skin permeability, we designed a solvent mixture system composed of ethanol, diethylene glycol monoethyl ether, cyclomethicone, and butylated hydroxyanisole, serving as a volatile solvent, nonvolatile co-solvent, spreading agent, and antioxidant, respectively. The ideal topical BIM formulation (BIM–TF#5) exhibited 4.60-fold higher human skin flux and a 529% increase in dermal drug deposition compared to BIM in ethanol. In addition, compared to the other formulations, BIM–TF#5 maximally activated human dermal papilla cell proliferation at a concentration of 5 μM BIM, equivalent to 10 μM minoxidil. Moreover, BIM–TF#5 (0.3% [w/w] BIM) significantly promoted hair regrowth in the androgenic alopecia mouse model and increased the area covered by hair at 10 days by 585% compared to the vehicle-treated mice, indicating that entire telogen area transitioned into the anagen phase. Furthermore, at day 14, the hair weight of mice treated with BIM–TF#5 (5% [w/w] BIM) was 8.45- and 1.30-fold greater than in the 5% (w/w) BIM in ethanol and 5% (w/v) minoxidil treated groups, respectively. In the histological examination, the number and diameter of hair follicles in the deep subcutis were significantly increased in the BIM–TF#5 (0.3 or 5% [w/w] BIM)-treated mice compared to the mice treated with vehicle or 5% (w/w) BIM in ethanol. Thus, our findings suggest that BIM–TF#5 is an effective formulation to treat scalp alopecia, as part of a novel therapeutic approach involving direct prostamide F2α receptor-mediated stimulation of dermal papilla cells within hair follicles.

## 1. Introduction

Alopecia refers to abnormal hair loss, typically accompanied by a change in the normal hair growth cycle (i.e. shortened anagen phase and prolonged catagen and telogen phases) (Qi & Garza, 2014). A variety of factors are associated with changes in the hair cycle, including hormonal imbalances, aging, autoimmune conditions, medications, nutritional deficiencies, and genetics (Houschyar et al., 2020). Although alopecia does not have severe physical symptoms, it does have significant adverse psychological and social effects (Saceda-Corralo et al., 2018). Therefore, treatment of alopecia is necessary to maintain the well-being of patients. Hair transplants and injection of growth factors and platelet-rich plasma are increasingly being used for alopecia patients, but have poor efficacy and the potential for various complications (Kerure & Patwardhan, 2018; Ro In et al., 2021). Treatments for alopecia aim to normalize the hair follicle size, density, and growth cycle.

The US Food and Drug Administration (FDA) has approved oral 5α-reductase inhibitors, including finasteride and dutasteride, and topical minoxidil, for immune (areata alopecia) as well as hormonal (androgenic alopecia) related alopecia, respectively. Finasteride is a synthetic 4-azasteroid compound that acts as a competitive and specific inhibitor of dihydrotestosterone receptors. Thus, it is contraindicated in women who are currently pregnant or hope to become pregnant in the future (Sallout & Al Wadi, 2009). In addition, many patients prefer to avoid oral medications due to possible adverse effects, such as decreased libido and erectile dysfunction in men (Kumar et al., 2011). Topical minoxidil is commonly used as an off-label treatment for male- and female-pattern baldness. Minoxidil increases blood flow to the hair follicles by dilating the arteries, thereby stimulating the production of growth factors from dermal papilla cells and activating the dermal papilla beta-catenin pathway, resulting in the induction and prolongation of the anagen phase, and stimulating the telogen-to-anagen transition (Choi et al., 2018). Topical minoxidil cannot prevent hair loss but may accelerate hair growth. Thus, it is not effective in all patients. Many patients consider topical minoxidil to be an outdated and ineffective treatment. Additionally, daily long-term use of minoxidil is poorly tolerated by patients as the hair becomes sticky and thus feels unclean; scalp irritation and itching also occur, mainly caused by propylene glycol (a key ingredient of topical minoxidil) (Suchonwanit et al., 2019; BinJadeed et al., 2021). Therefore, there is increasing demand for drugs that have few side effects and high efficacy.

Prostaglandins (PGs) are important modulators of hair growth. Therefore, PGE2 and PGF2α analogs and agonists could be useful for alopecia treatment. Bimatoprost (BIM; (Z)-7-[(1 R,2 R,3 R,5S)-3,5-dihydroxy-2-[(1E,3S)-3-hydroxy-5-phenyl-1-pentenyl] cyclopentyl]-5-N-ethylheptenamide) is a PGF2α analogue that stimulates eyelash growth when administered as 0.03% (w/v) ophthalmic solution for the management of ocular hypertension, which has led to FDA approval as a topical treatment of eyelash hypotrichosis (Carruthers et al., 2016). BIM stimulates hair growth by promoting the transition from the telogen to anagen phase, followed by prolongation of the anagen phase, thereby increasing hair length (Cohen, 2010; Tauchi et al., 2010). Moreover, it darkens the hair by promoting melanogenesis (Fagien, 2010), stimulates tyrosinase, and promotes melanin synthesis, dendricity of melanocytes, and melanosome conversion into keratinocytes. These effects are attributable to the action of BIM on specific receptors, such as PG and/or prostamide F2α receptors, on the cell membrane of the dermal papilla in the hair bulb. BIM stimulates intracellular signaling pathways and extracellular paracrine signaling, and also triggers changes in gene expression. Some paracrine signals arising from the dermal papilla cross the basement membrane to stimulate coordinated activity of keratinocytes and melanocytes, which increases hair growth and pigmentation (Khidhir et al., 2013). In addition, in a clinical trial, 0.03% (w/w) BIM was associated with the growth and development of eyebrow, eyelash, and hair follicles in cases of scalp alopecia areata. Briefly, 278 patients treated with 0.03% (w/w) BIM showed an average increase in their Global Eyelash Assessment score of one grade (Smith et al., 2012). Furthermore, in a 7-month phase III trial of 357 patients with hypotrichosis of the eyebrows, 0.03% (w/w) BIM dramatically increased eyebrow growth (Carruthers et al., 2016). In another trial, 0.03% (w/w) BIM application for 3 months decreased the severity of alopecia areata to a greater extent than the corticosteroid mometasone furoate (0.1% [w/w]) (Zaher et al., 2015). Based on these clinical trial results, BIM appears to be effective for hair regrowth in scalp alopecia.

Topical application is the most appropriate method for alopecia treatment because it limits potential side effects, such as unwanted hair growth in other areas, and interactions with other drugs. Although 0.03% (w/w) BIM solution has potential for the treatment of scalp alopecia, in phase II clinical trials it did not exhibit greater efficacy than topical minoxidil, suggesting that long-term treatment with a higher concentration is required to effectively treat scalp hair loss (Barrón-Hernández & Tosti, 2017). A topical BIM formulation with high drug content and enhanced infiltration thereof into the skin barrier may improve scalp hair regrowth.

To design the topical formulation, various delivery systems such as patches, pastes, creams, and ointments have been used (Garg et al., 2015). However, these all systems have several limitations. Patches have the disadvantages of a small application area and difficulty of application to the scalp (due to the presence of hair). Additionally, patches often irritate the skin because of their occlusive properties; the obstruction of sweat ducts prevents water vapor from evaporating off the skin surface. Patches are also difficult to apply to curved surfaces, cause pain when peeled off, and are esthetically unappealing. Semisolid preparations like creams, ointments, and pastes overcome some of these drawbacks but have other limitations, such as a lack of constant contact with the skin surface due to the formation of a rigid layer. Also, patient compliance is often poor because of the sticky and greasy feel of semisolid preparations (Kathe & Kathpalia, 2017). Therefore, a delivery system requiring less frequent dosing, due to constant close contact with the skin for a prolonged period of time, is needed to improve patient compliance. However, the rate and extent of drug absorption through the skin depends on the physiology thereof, as well as the physicochemical properties of the drug and delivery system. Interestingly, carrier systems such as ethosomes, liposomes, and transferosomes can enhance skin permeation (Zhang et al., 2014; Cristiano et al., 2020). In this study, we used a simple delivery system for volatile solvents, with the goal of overcoming the limitations of other systems. Our system can easily achieve supersaturation at the site of application by evaporating volatile solvents; this increases the drug concentration and effective solubility in the remaining vehicle on the skin (if nonvolatile solvents possess higher solubility of drug), thus enhancing skin penetration of the stratum corneum (Cilurzo et al., 2015).

The main objective of this study was to design a vehicle system of topical BIM, for instant drug delivery into hair follicles through the scalp of alopecia patients. Furthermore, we sought to determine the effects of daily topical administration of high-dose BIM on hair regrowth. This can be achieved by using a ‘solvent mixture system’ that includes ethanol, diethylene glycol monoethyl ether (Transcutol P), cyclomethicone, and butylated hydroxyanisole (BHA) for enhanced skin penetration and drug partitioning. This can serve as a volatile solvent, nonvolatile co-solvent, spreading agent, and antioxidant. The ideal composition of the BIM topical formulation (BIM–TF) was determined based on drug infiltration across an artificial membrane, as well as drug deposition and flux though the human skin. Thereafter, *in vitro* cell proliferation and recovery activities of the optimal BIM formulation on keratinocytes (HaCaT) and human follicle dermal papilla (hDP) cells in scratch wounds were evaluated. Finally, dose-dependent stimulation of hair regrowth, telogen hair coverage, hair weight, and the histology of hair follicles in the deep subcutis were assessed in an androgenic alopecia mouse model treated with BIM dissolved in ethanol, BIM–TFs, and commercial minoxidil topical solution.

## 2. Experimental design

### 2.1. Materials

BIM was purchased from Yonsung Fine Chemicals Co., Ltd. (Hwaseong-si, Republic of Korea). Glycerol, propylene glycol, octadec-9-enoic acid (oleic acid), polyethylene glycol 400 (PEG 400), polyoxyethylene (80) sorbitan monooleate (Tween 80), polyoxyethylene (20) sorbitan monolaurate (Tween 20), polyoxyethylene (20) oleyl ether (Brij O20), cyclomethicone, and BHA were purchased from Sigma-Aldrich Inc. (St. Louis, MO). Diethylene glycol monoethyl ether (Transcutol P) and propylene glycol monocaprylate (type II) (Capryol 90) were purchased from Gattefossé (Saint-Priest, France). Commercial 5% (w/v) minoxidil topical solution was obtained from Hyundai Pharmaceutical Co., Ltd. (Seoul, Republic of Korea). Other chemicals and solvents for high-performance liquid chromatography (HPLC) analyses were obtained from Merck (Darmstadt, Germany) and Thermo Fisher Scientific (Waltham, MA).

### 2.2. Animals

Male C57BL/6J mice (6–7 weeks old, 20–25 g) were purchased from G-Bio (Gwangju, Republic of Korea). The mice were housed in a standard environment with controlled temperature (23 ± 2 °C) and relative humidity (55 ± 10%), and a 12-h light/dark cycle. Animals were freely fed the standard laboratory diet (Nestlé Purina PetCare Company, St. Louis, MO) and ion-sterilized tap water. Ethical approval was obtained from the Institutional Animal Care and Use Committee (IACUC) of Mokpo National University (Mokpo, Republic of Korea; approval nos. MNU-IACUC-2021-014 and MNU-IACUC-2021-017). All animal experiments were performed in accordance with the National Institutes of Health Guidelines for the Care and Use of Laboratory Animals and IACUC guidelines.

### 2.3. Screening and selection of components for BIM–TF

Drug solubility is the most important factor for screening excipients for the supersaturated volatile vehicle system, to increase drug infiltration into the skin. An excess amount of BIM was added to 1 g of various hydroalcoholic solvents (ethanol, isopropyl alcohol, benzyl alcohol, glycerol, propylene glycol, and Transcutol P), lipophilic solvents (isopropyl myristate, oleic acid, and caprylic/capric triglyceride), and nonionic surfactants (PEG 400, Brij O20, Tween 20, Tween 80, and Capryol 90) in a sealed glass container. Mixtures of BIM and excipients were vortexed at 25 ± 1.0 °C in an isothermal shaker to reach equilibrium. After shaking for 48 h, the samples were centrifuged at 13,000 rpm for 15 min to remove excess drug, and the supernatant was further diluted with a mixture of water and acetonitrile (50:50, v/v). The BIM concentration in each solution was determined using an HPLC system with a Luna C18 column (4.6 × 250 mm, 5 µm, 100 Å) at 25 °C. An aliquot (30 µL) from each sample was eluted in the mobile phase: water with an 80–50% (v/v) linear gradient of acetonitrile for 5 min, followed by a 50–80% (v/v) linear gradient of acetonitrile for another 5 min at a flow rate of 1.0 mL/min. The limits of quantification and detection were determined as 0.05 and 0.025 µg/mL, respectively, at 205 nm using a UV–vis detector (model 2489; Waters, Milford, MA) (Annapurna et al., 2017); thus, the HPLC method was effective for quantifying the drug.

### 2.4. Preparation and *in vitro* artificial membrane permeability of BIM–TF

Ethanol was used as the solvent to prepare 5% (w/w) BIM–TF. For additional stability against drug oxidation, BHA was incorporated as an antioxidant in the drug formulation (Table 1). Briefly, 0.05 g of drug (5% [w/w] BIM based on 1 g solution) was weighed in a glass vial and dissolved in 0.3 g of ethanol. Then, 0.001 g of BHA was added, followed by vortex mixing. Afterwards, propylene glycol (0.22 g), oleic acid (0.05 g), or Transcutol P (0.22, 0.27, or 0.32 g) was added to the vial containing BIM, to improve the permeability of BIM across the skin. Then, cyclomethicone was added to the mixture at concentrations of 5% (w/w), 10% (w/w), and 15% (w/w) to improve its spreadability on the scalp. Moreover, 0.02, 0.04, and 0.08 g of water were incorporated into the formulation to promote skin hydration. The weight of the formulation was maintained at 1 g by adding ethanol (Table 1). Afterwards, the effects of different concentrations of various excipients in the vehicle system on drug solubility and miscibility with other components of BIM–TF were assessed. In addition, changes in drug content and physiochemical properties, including precipitation, re-crystallization, and color, were examined weekly for more than 3 months by visual inspection. Moreover, the supernatant was analyzed by HPLC after centrifugation, to assess the precipitation/recrystallization of the drug in the vehicle system. Finally, formulation color changes were assessed by UV–vis spectrophotometry (scanning range: 200–400 nm). The spectrum data on day 0 were used as the reference when estimating the color change according to the storage duration.

**Table 1.** Compositions and initial drug concentrations of topical BIM formulations.

Formulation (%, w/w)	BIM	Ethanol	Propylene glycol	Transcutol P	Oleic acid	Cyclomethicone	BHA	Water	Initial drug content (%)
BIM–TF#1	5	65.9	22		5		0.1	2	100 ± 3.18
BIM–TF#2	5	59.9	22		5		0.1	8	100 ± 2.37
BIM–TF#3	5	59.9		22	5		0.1	8	101 ± 2.35
BIM–TF#4	5	59.9		22		5	0.1	8	100 ± 5.72
BIM–TF#5	5	54.9		27		5	0.1	8	101 ± 1.47
BIM–TF#6	5	49.9		32		5	0.1	8	99.7 ± 2.69
BIM–TF#7	5	54.9		22		10	0.1	8	98.2 ± 1.33
BIM–TF#8	5	53.9		22		15	0.1	4	102 ± 1.00

BIM: bimatoprost; TF: topical formulation; BHA: butylated hydroxyanisole.

For preliminary comparison of the skin permeability of BIM among the vehicle systems, an *in vitro* artificial skin membrane permeability study was performed using the Franz diffusion cell system (Labfine, Gunpo, Republic of Korea). An artificial membrane (Strat-M^®^; EMD Millipore, Temecula, CA) was fixed between the donor and receptor phase compartments of a Franz diffusion cell with a diffusion area of 0.785 cm^2^. Then, the receptor compartment was filled with 5 mL of phosphate-buffered saline (PBS, pH 7.4). We assumed that sufficient sink conditions would be maintained due to the solubility of BIM in PBS (pH 7.4); 5.64 mg/mL of BIM was dissolved, which is 28.2-fold higher than the maximum drug concentration (200 µg/mL) in the receptor compartment. The solution in the receptor cell was stirred using a magnetic stirrer at 600 rpm, and a heating system was used to maintain the membrane surface temperature at 32 °C. Afterward, 20 µL of BIM–TF (1000 µg/cm^2^) was loaded into the donor compartment. Then, 200 µL of the receptor phase was withdrawn from each diffusion cell at pre-determined time points (0.5, 1, 1.5, 2, 3, 4, 5, 6, 8, and 10 hours) and the equivalent volume was replaced with fresh PBS (pH 7.4). To examine the effective permeation rate (flux), we used 10 h as the end point because permeation linearly increased up to, but not beyond, that time. The collected samples were filtered through a polyvinylidene fluoride (PVDF) membrane filter (0.45-µm pores) and the cumulative amount of BIM permeated through the artificial membrane was determined using the HPLC system at 205 nm, as described previously.

### 2.5. *In vitro* permeation and deposition of BIM in full-thickness human skin

To assess the infiltration of BIM–TF into human skin, cadaveric full-thickness skin from Caucasian thighs was purchased from a skin and tissue bank (HansBiomed Corp., Daejeon, Republic of Korea) and used as barrier membrane. *In vitro* skin permeability and deposition studies were performed using the Franz diffusion cell system. An excised full-thickness human skin sample was mounted on the receptor compartment of the diffusion cell, with the stratum corneum layer facing the donor cells. After assembling the donor compartment, the receptor compartment was filled with 5 mL of PBS (pH 7.4), stirred at 600 rpm, and heated to maintain a skin surface temperature of 32 °C. Prior to adding the sample solution, the diffusion cell system was allowed to equilibrate for more than 1 h to confirm the integrity of the epidermis, by ensuring transepidermal water loss (TEWL) <10 g/m^2^/h and transepithelial electrical resistance (TEER) of 1–2 kΩ (Zhang et al., 2018).

Next, 20 µL of each BIM–TF (1000 µg/cm^2^) was loaded into the donor compartment and 200 µL of the receptor phase was withdrawn from each diffusion cell at 0.5, 1, 1.5, 2, 3, 4, 5, 6, 8, and 10 hours. The removed volume was replaced with the same volume of fresh PBS (pH 7.4). After membrane filtering, permeated BIM in the sample solution was analyzed using the HPLC method, as described previously.

To evaluate drug deposition in the equilibrium state, as well as to estimate the effectiveness of once-a-day application, we analyzed drug deposition at 24 h after drug loading. The diffusion cell was disassembled, and the skin was carefully removed and washed three times with deionized water. After absorbing the residual water with a tissue paper, the skin exposed to the formulation was cut and weighed. In addition, to isolate the stratum corneum, skin was stripped 35 times with D-Squame (CuDerm, Dallas, TX) (Clausen et al., 2016). Next, the epidermis and dermis were separated by immersing the remaining skin in 5 mL of ice-cold 0.5% (w/v) dispase solution overnight (Macdiarmid & Wilson, 2001). After cutting the separated stratum corneum, epidermis, and dermis into small sections, the drug deposited in each layer was extracted with 2 mL of ethanol. The extracted sample solution was filtered using a 0.45-μm PVDF filter and dried in a centrifugal evaporator (Genevac Ltd., Ipswich, UK). Finally, the dried residue was reconstituted with 200 µL of ethanol; the BIM in each sample was determined at 205 nm using the HPLC system, as previously described, without any interference by the ethanol extract in the drug peak calculation.

### 2.6. *In vitro* cell proliferation assay

Cell proliferation assays were performed using HaCaT and hDP (PromoCell GmbH, Heidelberg, Germany) cells, to compare the biological activities of BIM–TF and minoxidil. Briefly, HaCaT or hDP cells were seeded to 96-well plates at a density of 2.5 × 10^3^ cells/well in 100 µL of Dulbecco’s modified Eagle’s medium (DMEM) containing 10% (v/v) fetal bovine serum (FBS) and 1% (v/v) penicillin/streptomycin or hDP medium containing 4% (v/v) of fetal calf serum, 4% (v/v) of bovine pituitary extract, 1 µg/L recombinant human fibroblast growth factor, and recombinant human insulin (5 mg/L) (PromoCell GmbH, Heidelberg, Germany). Then, the HaCaT and hDP cells were cultured at 37 °C in 95% air and 5% CO_2_ for 24 h. After incubation in serum-free medium for a further 24 h, the cells were treated with serially diluted BIM dissolved in ethanol, BIM–TF#1, and BIM–TF#5 (equivalent to 0.01, 0.1, 0.5, 1, 2.5, 5, and 10 μM BIM containing 0.000014, 0.00014, 0.0014, 0.0028, 0.007, 0.014, and 0.028% [v/v] ethanol, respectively), along with the respective concentrations of BIM–TF#5 vehicle solution and 10 μM of minoxidil solution containing 0.410% (v/v) ethanol. After incubation for an additional 48 h, cell viability was assessed using 10 μL of WST-1 reagent (Roche Diagnostics GmbH, Mannheim, Germany). The reagent was added to each well and incubated for 2 h. The absorbance at 450 nm was measured using a microplate reader (Multimode Plate Reader; PerkinElmer Inc., Waltham, MA). The percentages of viable cells in the treatment and untreated control groups were compared.

### 2.7. *In vitro* scratch-wound recovery assay

To investigate the cell proliferation and migration efficacies of BIM–TF in hDP cells, an *in vitro* scratch-wound recovery assay was performed. We added 2.5 × 10^4^ hDP cells to each well in 1 mL of hDP medium containing 4% (v/v) of fetal calf serum, 4% (v/v) of bovine pituitary extract, 1 µg/L recombinant human fibroblast growth factor, and recombinant human insulin (5 mg/L) on a collagen-coated 12-well plate. The plates were incubated at 37 °C for 72 h to produce a confluent monolayer. Then, a cell-free scratch was created using a sterilized micro-pipette tip and the detached cells from each monolayer were washed three times with PBS (pH 7.4). hDP cells were further treated with 1 mL of BIM dissolved in ethanol, BIM–TF#1, BIM–TF#5, and vehicle of BIM–TF#5 diluted with hDP medium containing 0.5% (v/v) fetal calf serum (equivalent to 5 μM BIM) or 10 μM of minoxidil in hDP medium containing 0.5% (v/v) fetal calf serum. The plate with the hDP cells was incubated at 37 °C and wound recovery was observed using a microscope at 0, 6, 12, 24, and 48 h after the treatment.

### 2.8. *In vivo* hair regrowth efficacy of BIM–TF

To evaluate the hair regrowth efficacy of BIM–TF, androgenic alopecia was induced in C57/BL6 mice by once-daily topical treatment of dihydrotestosterone (0.5 mg/kg) for five days, and the dorsal hair of mice were shaved using animal clippers and duct tape (Fu et al., 2021). Then, the mice were randomly divided into the following seven groups (20 mice/group) for topical treatment: control (vehicle) (vehicle solution of BIM–TF#5), minoxidil topical solution (5%) (commercial 5% [w/v] minoxidil topical solution), BIM in ethanol (5%) (5% [w/w] BIM in ethanol), BIM–TF#1 (5%) (BIM–TF#1 containing 5% [w/w] BIM), BIM–TF#5 (0.03%) (BIM–TF#5 containing 0.03% [w/w] BIM), BIM–TF#5 (0.3%) (BIM–TF#5 containing 0.3% [w/w] BIM), and BIM–TF#5 (5%) (BIM–TF#5 containing 5% [w/w] BIM). Additionally, 20 control mice were shaved with clippers and treated with normal saline. We applied 20 µL of each sample solution to the dorsal skin once per day for 14 days. Hair regrowth was examined in dorsal photographs taken 0, 3, 5, 7, 10, and 14 days after treatment. The rate of hair growth was estimated using ImageJ software (NIH, Bethesda, MD) based on the length of hair follicles, area covered with hair, areas of conversion from telogens to anagens (black skin), and remaining areas of telogens (pink skin). Fourteen days after treatment, hair in the treatment area was measured by cutting the regrown hair and weighing it in an analytical balance (Orasan et al., 2016). In addition, 0, 3, 7, 10, and 14 days after treatment, four mice per group were sacrificed, and the dorsal skin tissues were excised and immediately fixed in 10% (v/v) neutral phosphate-buffered formalin. The skin samples were processed using routine procedures and embedded in paraffin wax. The tissues were cut into 5-μm-thick sections and stained with hematoxylin and eosin (H&E) to assess gross histopathological changes and examine hair follicles in the treated area.

### 2.9. Statistical analysis

All data are expressed as mean ± standard deviation (SD) or standard error of the mean (SEM). *p* Values less than .05 were considered statistically significant. One-way analysis of variance (ANOVA) followed by Tukey’s multiple comparison test was performed for analysis of three or more mean values.

## 3. Results and discussion

### 3.1. Selection of excipients and preparation of BIM–TF

**Figure 1.**
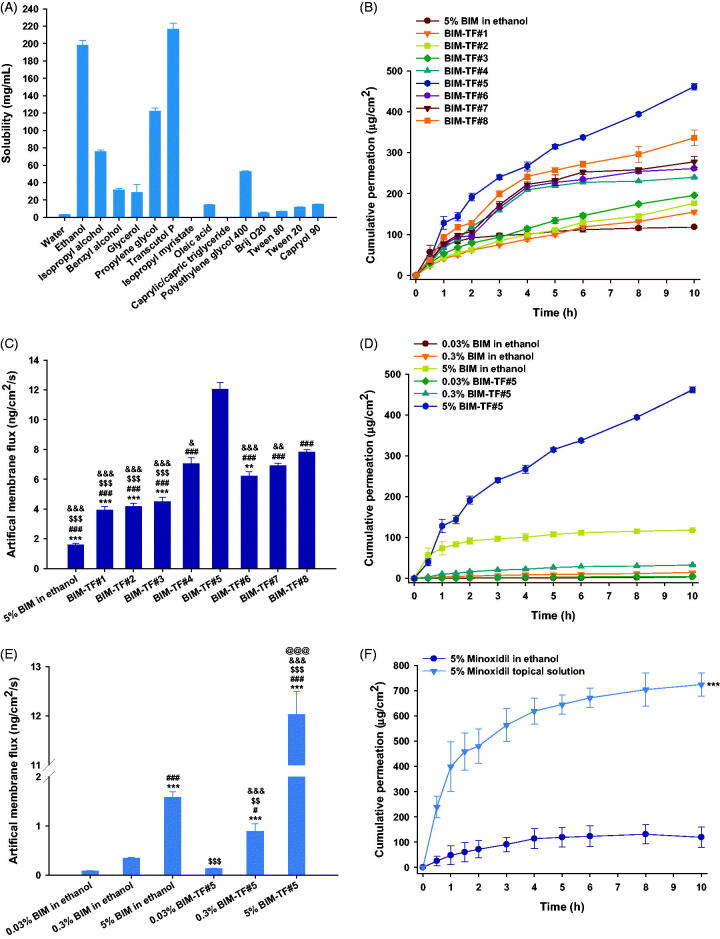
(A) Solubility of bimatoprost (BIM) in various solvents and surfactants. *In vitro* penetration of BIM topical formulations (BIM–TFs) through the artificial membrane; (B) time–course curve showing cumulative drug infiltration and (C) flux from BIM in ethanol or BIM–TFs. **p<.01, ***p<.001 compared to BIM–TF#4; ^###^*p*<.001 compared to BIM–TF#5; ^$$$^*p*<.001 compared to BIM–TF#7; ^&^*p*<.05, ^&&^*p*<.01, ^&&&^*p*<.001 compared to BIM–TF#8. (D) Time–course curve showing cumulative infiltration and (E) flux of 0.03%, 0.3%, and 5% BIM in ethanol or BIM–TF#5. ***p<.001 compared to 0.03% BIM in ethanol; ^#^*p*<.05, ^###^*p*<.001 compared to 0.3% BIM in ethanol; ^$$^*p*<.01, ^$$$^*p*<.001 compared to 5% BIM in ethanol; ^&&&^*p*<.001 compared to 0.03% BIM–TF#5; ^@@@^*p*<.001 compared to 0.3% BIM–TF#5. (F) Time–course curve showing cumulative drug penetration of 5% minoxidil in ethanol and commercial 5% minoxidil topical solution through an artificial membrane. ***p<.001 compared to 5% minoxidil in ethanol. Values are means ± SDs (*n* = 4 for each group).

As maintaining of supersaturation at the site of application after evaporation of volatile solvents can significantly increase the thermodynamic driving force above unity, a solvent system that maintains drug solubility during evaporation of the volatile solvent and infiltration through the dermis can influence the thermodynamic driving force for scalp tissue permeation after topical application (Surber & Knie, 2018). Although BIM is highly soluble in alcohol, fast evaporation of the topical solution can interfere with skin infiltration due to precipitation of the drug molecules on the skin surface before absorption. Thus, it is challenging to dissolve the drug to therapeutically effective levels in a topical formulation composed of volatile and/or nonvolatile solvents with enhancers. Therefore, the solubility of BIM in various excipients, including water, hydroalcoholic solvents, lipophilic solvents, and nonionic surfactants, was assessed (Figure 1(A)). Among the alcohols, the solubility of BIM in ethanol was 198 ± 5.40 mg/mL, which was 56.8-, 2.61-, and 6.31-fold higher than that in water, isopropyl alcohol, and benzyl alcohol, respectively. As BIM consists of polar (amide side chain) and non-polar (pentene ring with hydroxyl group) groups, it can be dissolved in both phases. However, the dominance of the polar group of BIM may explain its higher solubility in ethanol, which is more polar than benzyl alcohol but less polar than water and isopropyl alcohol. In addition, BIM was less soluble in lipophilic solvents, such as oleic acid and isopropyl myristate, and nonionic surfactants, including Brij O20, Tween 80, Tween 20, and Capryol 90. However, the solubility of BIM in propylene glycol and Transcutol P was 122 ± 3.55 and 217 ± 6.83 mg/mL, respectively. Transcutol P can dissolve a wide range of hydrophilic and lipophilic substances. Its higher solubilization power than propylene glycol and ethanol makes it a highly useful pharmaceutical excipient (Osborne, 2011). Transcutol P has a log *P* of almost −0.43; therefore, it is considered a polar protic solubilizer with good affinity and miscibility with hydrophobic groups (Osborne & Musakhanian, 2018). Furthermore, Transcutol P is compatible with most pharmaceutical excipients, soluble in common solvents such as propylene glycol, ethanol, and water, and miscible with polar lipids such as medium-chain triglycerides and PEG-based surfactants (Ganem-Quintanar et al., 1997).

Thus, based on the solubility data, ethanol and Transcutol P were selected as the volatile solvent and nonvolatile co-solvent, respectively, for preparation of an effective topical vehicle system for BIM (Mura et al., 2000). In addition, BIM–TF#1 was produced as a reference formulation to compare its miscibility and skin absorption properties with those of propylene glycol. Furthermore, cyclomethicone was incorporated into the formulations as a wetting agent due to its varying evaporation rates and non-greasy texture (Houben, 2020). To demonstrate effective permeability into the skin, skin hydration should be maintained (Tan et al., 2010). Therefore, deionized water was added to maintain sufficient skin hydration. However, all formulations led to an approximately 20% decrease in drug content after 21 days, indicating oxidative degradation of BIM during storage. To overcome this problem, we added BHA into the formulations as an antioxidant, which maintained 95–105% of the drug content in each vehicle system for more than 3 months at room temperature. In addition, there were no changes in physical appearance with the addition of BHA, such as precipitation, phase separation, or color change.

### 3.2. *In vitro* artificial membrane permeability of BIM–TFs

The permeability through the artificial membrane of various formulations of BIM was determined using the Franz diffusion cell system. Drug infiltration of BIM–TF#1 and BIM–TF#2 through the artificial membrane improved by 1.31- and 1.49-fold, respectively, compared to that of BIM–TF in ethanol (Figure 1(B)). The permeation of BIM from BIM–TF#3 up to 10 h was found to be 10.8% higher than that of BIM–TF#2, suggesting an effective role of Transcutol P in the enhancement of drug permeability through the artificial skin membrane. However, when cyclomethicone was incorporated into the BIM–TF#4 instead of oleic acid, the permeated BIM at 10 h was significantly increased, by 22.6%, compared to that of BIM–TF#3, which may be attributed to improved wettability of the formulation on the membrane (Figure 1(B)). Furthermore, when the Transcutol P concentration was increased by 5% (w/w) in BIM–TF#4 (i.e. BIM–TF#5), the permeability of BIM up to 10 h was significantly increased, by 92.3%, compared to that of BIM–TF#4, resulting in 7.61-, 3.07-, and 1.71-fold greater flux than BIM in ethanol, BIM–TF#1, and BIM–TF#4, respectively (Figure 1(C)). However, no further penetration-enhancing effect was achieved by increasing the concentrations of Transcutol P or cyclomethicone, which may be due to a decrease in drug partition into the skin caused by the water absorption properties of Transcutol P, resulting in changes in drug solubility and thermodynamic activity (Björklund et al., 2016; Subedi et al., 2021).

In addition, the cumulative drug permeation achieved 10 h after loading 0.03% (w/w) of BIM–TF#5 was 4.94 ± 0.197 μg/cm^2^, representing a 56.5% higher flux value compared to that of 0.03% (w/w) BIM in ethanol (Figure 1(D,E)). In addition, a 10-fold increase in the drug concentration of BIM–TF#5 (from 0.03% [w/w] to 0.3% [w/w]) yielded a flux value of 0.891 ± 0.154 ng/cm^2^/s, which was 2.60-fold greater than that of 0.3% (w/w) BIM in ethanol (Figure 1(E)). Furthermore, increasing the BIM concentration in BIM–TF#5 from 0.3% (w/w) to 5% (w/w) increased the cumulative permeated drug amount for 10 h and flux by 14.0- and 13.5-fold, respectively, implying that the drug penetration from BIM–TF#5 was increased in a concentration-dependent manner without any decrease in the thermodynamic driving force for permeability, due to drug precipitation at high concentrations.

Importantly, the cumulative drug amount from the commercialized 5% (w/v) minoxidil topical solution that permeated the artificial membrane was 725 ± 45.2 µg/cm^2^ at 10 h, which was 6.08-fold higher than that of 5% (w/w) minoxidil in ethanol, resulting in a 259% increase in flux compared to that of 5% (w/w) minoxidil ethanol solution (Figure 1(F)).

### 3.3. *In vitro* human skin permeability and deposition of BIM-TFs

**Figure 2.**
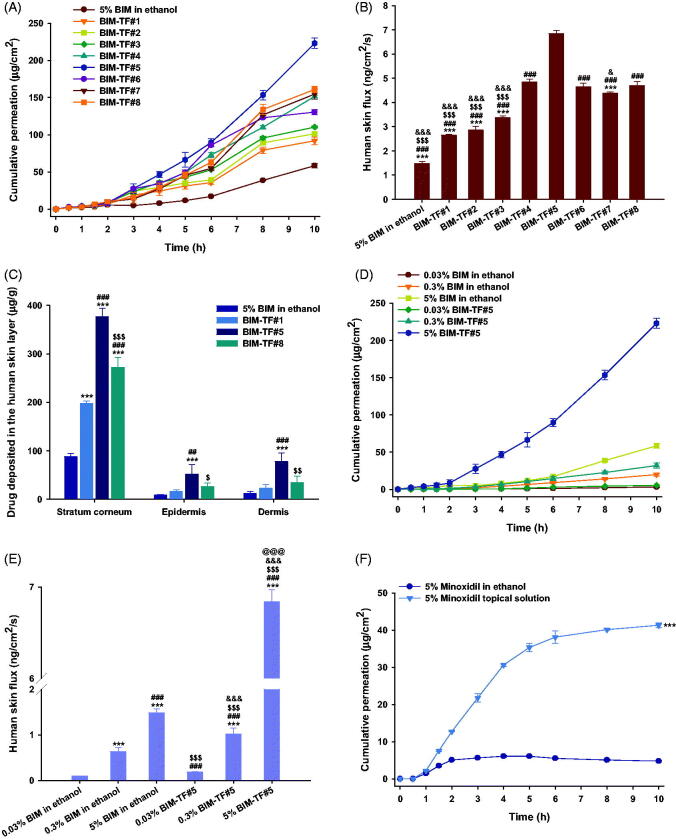
*In vitro* full-thickness human skin permeability of bimatoprost (BIM) in ethanol and topical formulations (BIM–TFs). (A) Time–course curve showing cumulative levels of BIM infiltration through the human skin and (B) flux from 5% BIM in ethanol and BIM–TFs over 10 h. ***p<.001 compared to BIM–TF#4; ^###^*p*<.001 compared to BIM–TF#5; ^$$$^*p*<.001 compared to BIM–TF#6; ^&^*p*<.05, ^&&&^*p*<.001 compared to BIM–TF#8. (C) BIM deposited in 1 g of human stratum corneum, epidermis, and dermis from 5% BIM in ethanol and BIM–TFs for 24 h. ***p<.001 compared to 5% BIM in ethanol; ^##^*p*<.01, ^###^*p*<.001 compared to BIM–TF#1; ^$^*p*<.05, ^$$^*p*<.01, ^$$$^*p*<.001 compared to BIM–TF#5. (D) Time–course curve of cumulative BIM infiltration and (E) flux through human skin of 0.03%, 0.3%, and 5% BIM in ethanol and BIM–TF#5 for 10 h. ***p<.001 compared to 0.03% BIM in ethanol; ^###^*p*<.001 compared to 0.3% BIM in ethanol; ^$$$^*p*<.001 compared to 5% BIM in ethanol; ^&&&^*p*<.001 compared to 0.03% BIM–TF#5; ^@@@^*p*<.001 compared to 0.3% BIM–TF#5. (F) Time–course curve showing cumulative BIM concentrations permeating human skin from 5% minoxidil in ethanol and commercial 5% minoxidil topical solution over 10 h. ***p<.001 compared to 5% minoxidil in ethanol. Values are means ± SDs (*n* = 4 for each group).

The *in vitro* permeation of BIM in ethanol and BIM–TFs of human skin was evaluated. At 10 h after drug loading, the cumulative permeated BIM was 73.5% higher for BIM–TF#2 compared to BIM in ethanol, resulting in a 92.5% increase in skin flux of BIM in ethanol (Figure 2(A,B)). However, skin permeation of BIM from BIM–TF#2 was not significantly improved by replacing the co-solvent propylene glycol with Transcutol P (i.e. BIM–TF#3). The cumulative BIM permeation from BIM–TF#4 at 10 h was significantly increased by replacing the oleic acid with cyclomethicone, leading to a 37.3% higher value than for BIM–TF#3 (Figure 2(A)). Cyclomethicone enhances skin moisture retention, and acts synergistically with Transcutol P to promote intercellular lipid fluidization in stratum corneum (Purnamawati et al., 2017). Moreover, after increasing the concentration of Transcutol P in BIM–TF#4 from 22% (w/w) to 27% (w/w), BIM–TF#5 exhibited a 1.47-fold increase in drug infiltration into human skin after 10 h compared to BIM–TF#4, resulting in a 360% increase in BIM flux in ethanol (Figure 2(A,B)). However, the permeated drug levels for BIM–TF#6 and BIM–TF#7 at 10 h were 41% and 31% lower than that for BIM–TF#5, respectively (Figure 2(A,B)). These results are in agreement with the *in vitro* artificial membrane permeability data and may be explained by reduced drug partitioning in the skin. Transcutol P and cyclomethicone may optimize drug and vehicle properties, and modify the stratum corneum by increasing the thermodynamic driving force, thereby enhancing drug solubility/partitioning, maintaining hydration, and causing intercellular lipid fluidization.

The BIM deposition levels in various skin layers after application of BIM in ethanol, BIM–TF#1, BIM–TF#5, and BIM–TF#8 for 24 h are displayed in Figure 2(C). After topical application of BIM in ethanol, drug deposition was highest in the stratum corneum, followed by the dermis and epidermis, presumably because of low aqueous solubility of BIM. Therefore, the drug remains in the lipids of the stratum corneum after evaporation of ethanol (Kwak et al., 2012). Dermal deposition from the propylene glycol-based formulation (i.e. BIM–TF#1) was 1.81-fold higher than that from BIM in ethanol. Moreover, from the Transcutol-based vehicle BIM–TF#8, drug deposition in the stratum corneum, epidermis, and dermis was significantly increased, by 37.8%, 65.3%, and 52.7%, respectively, compared to BIM–TF#1. Transcutol readily permeates the intercellular spaces and alters the solubility of intercellular lipid domains to match the solubility parameters typical of pharmaceutical actives. This in turn enhances drug solubility, penetration, and permeation into the stratum corneum (Otto et al., 2008). BIM–TF#5 demonstrated 1.38- and 2.27-fold greater stratum corneum and dermal drug infiltration compared to BIM–TF#8, which may reflect more effective drug partitioning in the human skin from BIM–TF#5. Although Transcutol P readily penetrates the stratum corneum and strongly binds with the intercellular water to modify skin permeation of the active ingredient, it also significantly contributes to swelling of the intercellular spaces of the stratum corneum. This unique ability to cause swelling of the intercellular spaces in the skin barrier can cause skin retention of the drug, depending on its molecular structure and aqueous solubility (Ritschel et al., 1991). BIM–TF#5 has the ideal composition of Transcutol P and cyclomethicone for topical delivery of BIM, which can more effectively stimulate the hair follicles in the deep dermal layer of the scalp than other topical formulations.

Similarly, at all drug levels, BIM–TF#5 significantly increased drug absorption through human skin compared to BIM dissolved in ethanol (Figure 2(D)). Moreover, as the drug concentration in BIM–TF#5 was increased from 0.03% (w/w) to 0.3% (w/w), human skin flux was increased by 5.42-fold, and a further increase in the drug concentration of BIM–TF#5 to 5% (w/w) was associated with a 6.74-fold increase in skin flux compared to 0.3% (w/w) BIM–TF#5. Therefore, drug absorption through the human skin from BIM–TF#5 was proportionally increased with increasing drug concentration in the formulation.

Furthermore, the commercial 5% (w/v) minoxidil topical solution also demonstrated 88.4% higher cumulative drug permeation over 10 h compared to 5% (w/w) minoxidil in ethanol, which may be due to the presence of propylene glycol in 5% (w/v) minoxidil topical solution (Figure 2(F)). Thus, the skin flux of 5% (w/v) minoxidil topical solution was 2.02 ± 0.064 ng/cm^2^/s, which was 4.81-fold greater than that of 5% (w/w) minoxidil in ethanol, but 3.39-fold lower than that of 5% (w/w) BIM–TF#5.

Based on the *in vitro* skin permeation and flux data, BIM–TF#5 was identified as the ideal topical BIM formulation. Further studies, for example, the *in vitro* cell proliferation activity and *in vivo* hair regrowth effects of topical BIM, in androgenic alopecia-induced mice were carried out using BIM–TF#5.

### 3.4. Promotion of HaCaT and hDP cell proliferation

**Figure 3.**
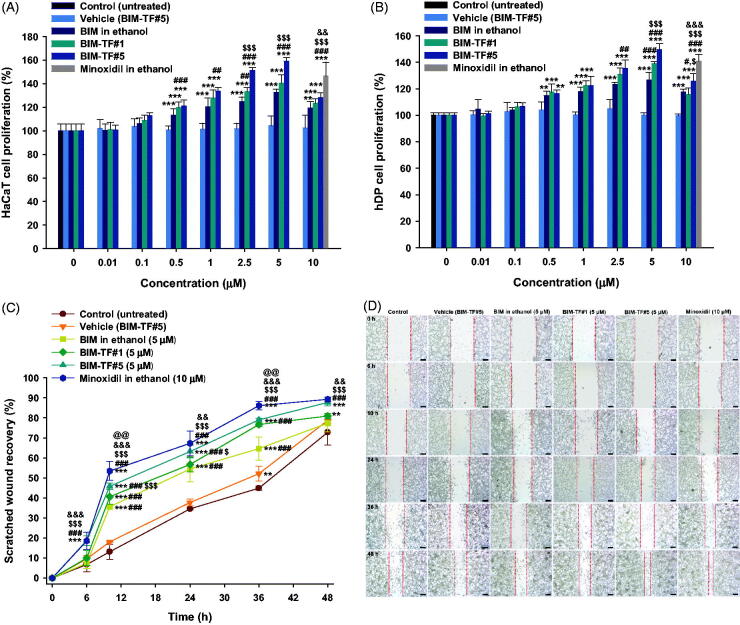
Relative cell proliferation of (A) keratinocyte (HaCaT) and (B) human follicle dermal papilla (hDP) cells after incubation with bimatoprost (BIM) in ethanol, BIM topical formulations (BIM–TFs), and vehicle (BIM–TF#5 diluted to BIM concentrations of 0.01, 0.1, 0.5, 1, 2.5, 5, and 10 µM BIM), and 5% minoxidil in ethanol diluted to a concentration equivalent to that of 10 µM minoxidil. **p<.01, ***p<.001 compared to vehicle (BIM–TF#5); ^#^*p*<.05, ^##^*p*<.01, ^###^*p*<.001 compared to BIM in ethanol; ^$^*p*<.05, ^$$$^*p*<.001 compared to BIM–TF#1; ^&&^*p*<.01, ^&&&^*p*<.001 compared to BIM–TF#5. Relative scratch wound recovery of hDP cells after incubation with BIM in ethanol, BIM–TF#1, BIM–TF#5, and vehicle of BIM–TF#5 diluted to a concentration equivalent to that of 5 μM BIM, and minoxidil in ethanol diluted to a concentration equivalent to that of 10 µM minoxidil. (C) Time–course curve of relative scratch wound recovery area of hDP cells. **p<.01, ***p<.001 compared to control (untreated); ^###^*p*<.001 compared to vehicle (BIM–TF#5); ^$^*p*<.05, ^$$$^*p*<.001 compared to BIM in ethanol (5 µM); ^&&^*p*<.01, ^&&&^*p*<.001 compared to BIM–TF#1 (5 µM); ^@@^*p*<.01 compared to BIM–TF#5 (5 µM). Values represent means ± SDs (*n* = 6). (D) Representative microscope images of scratch wounds of hDP cells. Scale bar = 200 µm.

To assess the biological activity of BIM after incorporating it into BIM–TF#5, we performed a cell proliferation assay using HaCaT and hDP cells; vehicle (BIM–TF#5), BIM in ethanol, BIM–TF#1, BIM–TF#5, and 10 µM minoxidil were compared. As shown in Figure 3(A,B), cell viability was maintained at 100% even after vehicle (BIM–TF#5) treatment, indicating no toxicity of the vehicle. The effect of drug on cell proliferation was dose-dependent and increased from 0.01 to 5 µM. However, increasing the concentration of BIM to 10 µM did not further increase cell proliferation, probably due to saturation of the receptor-mediated effects, as reported in other tissues (Khidhir et al., 2013), and increase of ethanol in the treatment medium as the drug concentration increased. Briefly, when the HaCaT cells were treated with 5 µM of BIM from BIM–TF#5 for 48 h, the cell proliferation value was reached to 159%, which was 26.6%, 18.74%, and 12.4% higher compared to BIM in ethanol (5 µM), BIM–TF#1 (5 µM), and minoxidil (10 µM), respectively (Figure 3(A)). Furthermore, the trend of cellular proliferation in hDP cell was also similar as like in HaCaT cell. After treatment of hDP cells with 5 µM of BIM from BIM in ethanol for 48 h showed 26.8% more cell proliferation than untreated cells (Figure 3(B)). In addition, the treatment of hDP cell with 5 µM of BIM from the reference formulation (BIM–TF#1) containing propylene glycol and oleic acid as a co-solvent and surfactant, respectively, demonstrated 12.2% higher cell proliferation compared to BIM in ethanol as well as equivalent to cellular proliferation of the minoxidil (10 µM) treated group (Figure 3(B)). Furthermore, the 48-h incubation with 5 µM of BIM–TF#5 activated cellular proliferation to 150% which was 49.5%, 10.5%, and 8.82% higher than those of the vehicle, BIM–TF#1 (5 µM), and minoxidil (10 µM), respectively. This enhanced cell proliferation of BIM in both keratinocytes and human dermal papilla cells after incorporating it into BIM–TF#5 may be due to the presence of Transcutol P and cyclomethicone which facilitated partitioning of drug toward the cell membrane, resulting in sufficient binding of drug molecule with the receptor present on the cell membrane (Varis et al., 2000; Spósito et al., 2017). Greater solubility of BIM in Transcutol P may be another reason for the high cellular uptake of the drug from BIM–TF#5, resulting in enhanced cellular proliferation. Thus, the high cell proliferation activity of BIM–TF#5 on keratinocytes and human dermal papilla cells may lead to potent rejuvenation and stimulation of hair follicles, and thus to hair development (i.e. a prolonged anagen phase).

To further evaluate the effect of the formulations on cellular activities, *in vitro* wound recovery assay was performed using hDP cells for 48 h. The recovery rate of wounds treated with vehicle (BIM–TF#5) was similar to that of cells treated with the control, which accords with the results of the cell proliferation assay; i.e. there was no inhibitory effect on cell migration by the vehicle (BIM–TF#5). The wound recovery rate in hDP cells was accelerated after treatment with BIM compared to the control (untreated) and vehicle (BIM–TF#5). As shown in Figure 3(C,D), the wound closure areas after 36-h incubation with BIM in ethanol (5 µM) and BIM–TF#1 (5 µM) in hDP scratch were 19.7% and 31.8% larger than in the control (untreated) group, respectively; the wound recovery areas at 36 h were 64.6% and 76.7%, respectively. At 48 h after treatment with 5 µM of BIM from BIM–TF#5, the wound closure area increased to 87.7% which was 10.3% and 6.77% larger than those for BIM in ethanol and BIM–TF#1, respectively. In addition, the wound closure areas after 48-h treatment with BIM-TF#5 (5 µM) and minoxidil (10 µM) were similar. Thus, BIM–TF#5 (5 µM BIM) significantly accelerated wound recovery compared to BIM in ethanol and BIM–TF#1, with a largely similar wound closure rate to that of minoxidil (10 µM) in the hDP cells. This confirmed that BIM–TF#5 is an effective formulation for the treatment of alopecia via biological activation of hair follicle cells.

### 3.5. *In vivo* hair regrowth effects of BIM–TF

#### 3.5.1. Stimulation of telogen hair coverage

**Figure 4.**
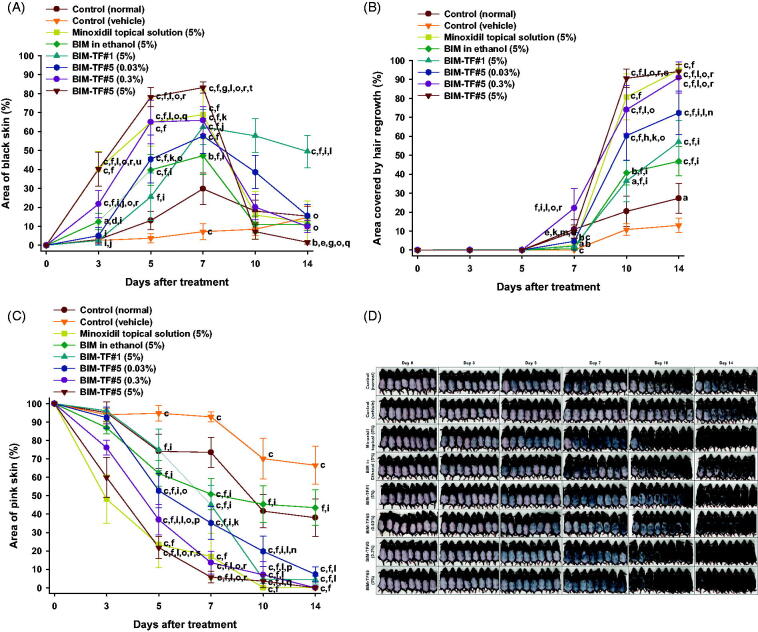
*In vivo* hair regrowth efficacy of once-daily bimatoprost topical formulations (BIM–TFs) and 5% minoxidil topical solution in androgenic alopecia mice. (A) Time–course curve of changes in dorsal skin color from pink to black, indicating hair regrowth (anagen). (B) Time–course curve of hair growth on dorsal skin. (C) Time–course curve of dorsal skin showing a pink color (telogen). ^a^*p*<.05, ^b^*p*<.01, ^c^*p*<.001 compared to control (normal); ^d^*p*<.05, ^e^*p*<.01, ^f^*p*<.001 compared to control (vehicle); ^g^*p*<.05, ^h^*p*<.01, ^i^*p*<.001 compared to minoxidil topical solution (5%); ^j^*p*<.05, ^k^*p*<.01, ^l^*p*<.001 compared to BIM in ethanol (5%); ^m^*p*<.05, ^n^*p*<.01, ^o^*p*<.001 compared to BIM–TF#1 (5%); ^p^*p*<.05, ^q^*p*<.01, ^r^*p*<.001 compared to BIM–TF#5 (0.03%); ^s^*p*<.05, ^t^*p*<.01, ^u^*p*<.001 compared to BIM–TF#5 (0.3%). Values are means ± SEMs (*n* = 8 for each group). (D) Representative photographs of mice dorsal skin showing hair regrowth at 0, 3, 5, 7, 10, and 14 days after treatment.

To evaluate the hair growth efficacy of alopecia mice after application of BIM–TF at various concentrations, dorsal skin areas that appeared black (anagen) or were covered by regrown hair, and remaining pink skin areas (telogen), were measured on treatment days 0, 3, 5, 7, 10, and 14 (Figure 4). The hair follicles in the telogen phase were significantly increased in the vehicle-treated mice compared to the controls. For up to 14 days after treatment, the complete hair regrowth area in the vehicle mice was only 13.0 ± 3.73%, which was 2.10-fold lower than that observed in normal shaved mice, suggesting that androgenic alopecia was successfully induced in mice by topical application of dihydrotestosterone (0.5 mg/d). The latency to a color change of the treatment area from pink to black, indicating the early anagen phase, was recorded. The average latency for all mice in each group was calculated. For the 5% (w/v) minoxidil topical solution-treated group, the latency was 4.13 ± 2.47 days, which was 2.56- and 1.79-fold faster compared to the normal and vehicle treated groups, respectively; 80.8 ± 12.1% of the skin in the telogen area was covered by new hair at 10 days after treatment (Figure 4(A,B)). Thus, once-daily topical treatment with 5% (w/v) minoxidil solution significantly promoted new hair growth in androgenic alopecia mice. When the mice were treated with once-daily BIM in ethanol (5%, w/w) or BIM–TF#1 (5%, w/w) for 14 days, the hair regrowth areas were 46.9%±7.71% and 57.0 ± 11.2%, respectively, i.e. were significantly larger than in the control (vehicle) group; however, they were 2.02- and 1.66-fold smaller than for the minoxidil topical solution (5%, w/v) (Figure 4(A,B)). BIM–TF#5 application for 14 days significantly accelerated the next hair cycle at all concentrations, with new hair growth clearly visible on day 7 in some mice. Mice treated with BIM–TF#5 (0.03% [w/w] or 0.3% [w/w]) exhibited an early change in skin color to black, at 4.50 ± 1.14 and 3.50 ± 0.93 days, respectively (Figure 4(A)). Moreover, on day 3, 21.8 ± 7.20% of dorsal skin displayed signs of anagens in the BIM–TF#5 (0.3%, w/w) treatment group, with these skin areas being 733%, 73.8%, and 753% larger than those of the control (vehicle), BIM in ethanol (5%, w/w), and BIM–TF#1 (5%, w/w) treatment groups, respectively (Figure 4(A)). At seven days after treatment, the areas covered by regrown hair were significantly larger in the 0.03% (w/w) and 0.3% (w/w) BIM–TF#5 treatment groups (4.62 ± 4.62% and 22.1 ± 10.4%, respectively) than the vehicle (0%, w/w) and 5% (w/w) BIM in ethanol (0.788 ± 0.577%) groups (Figure 4(B)). Maximum hair regrowth in the BIM–TF#5 (0.3%, w/w) group was seen on day 10, with 74.1 ± 12.8% hair coverage of dorsal telogen skin, which was much higher compared to the BIM–TF#1 (5%, w/w) group (36.4 ± 10.8%) and equivalent to that in the 5% (w/v) minoxidil topical solution group (80.8 ± 12.1%) (Figure 4(A,B)).

Furthermore, BIM–TF#5 exhibited concentration-dependent stimulation of hair growth. Within the first three days after treatment with BIM–TF#5 (5%, w/w), the skin color changed from pink to black; the rate of change was significantly faster than in mice treated with the vehicle (control), BIM in ethanol (5%, w/w), and BIM–TF#5 (0.3%, w/w) (Figure 4(C)). In addition, hair growth in the androgenic area treated with BIM–TF#5 (5%, w/w) was greater at day 3 compared to the BIM in ethanol (5%, w/w) and BIM–TF#1 (5%, w/w) treatment groups (Figure 4(B)). At 10 days after treatment, mice treated with BIM–TF#5 (5%, w/w) showed 738%, 123%, and 12.1% greater hair regrowth in the androgenic area than the control (vehicle), BIM in ethanol (5%, w/w), and minoxidil topical solution (5%, w/v) groups, respectively (Figure 4(B)). However, stimulation of hair regrowth by BIM–TF#5 (5%, w/w) from 10 days after treatment was similar to that in the BIM–TF#5 (0.3%, w/w) treatment group (Figure 4(B)), indicating saturation of a receptor-mediated effect on hair growth. The remaining telogen area sizes at day 10 were 70.1 ± 11.0% for the control (vehicle) group, 45.4 ± 10.0% for the BIM in ethanol (5%, w/w) group, 0.163 ± 0.163% for the minoxidil topical solution (5%, w/v) group, 4.73 ± 2.56% for the BIM–TF#1 (5%, w/w) group, 19.8 ± 8.16% for the BIM–TF#5 (0.03%, w/w) group, 7.08 ± 7.08% for the BIM–TF#5 (0.3%, w/w) group, and 3.63 ± 3.63% for the BIM–TF#5 (5%, w/w) group (Figure 4(C)). At day 14, dorsal skin treated with 0.3% (w/w) or 5% (w/w) BIM–TF#5 or 5% (w/v) minoxidil topical solution was completely covered by new hair (91.0 ± 8.16%, 94.2 ± 3.56%, and 94.8 ± 3.51%, respectively), whereas 38.1 ± 10.3%, 66.5 ± 10.3%, and 43.5 ± 9.66% of skin remained in the telogen state in the control (normal), control (vehicle), and BIM in ethanol (5%) groups, respectively (Figure 4(D)). These results suggest that enhanced topical delivery of BIM can reduce the time spent by hair follicles in the telogen stage in androgenetic alopecia, and accelerates the transition to the anagen phase to reinitiate hair growth.

#### 3.5.2. Hair weight and histological evaluation

**Figure 5.**
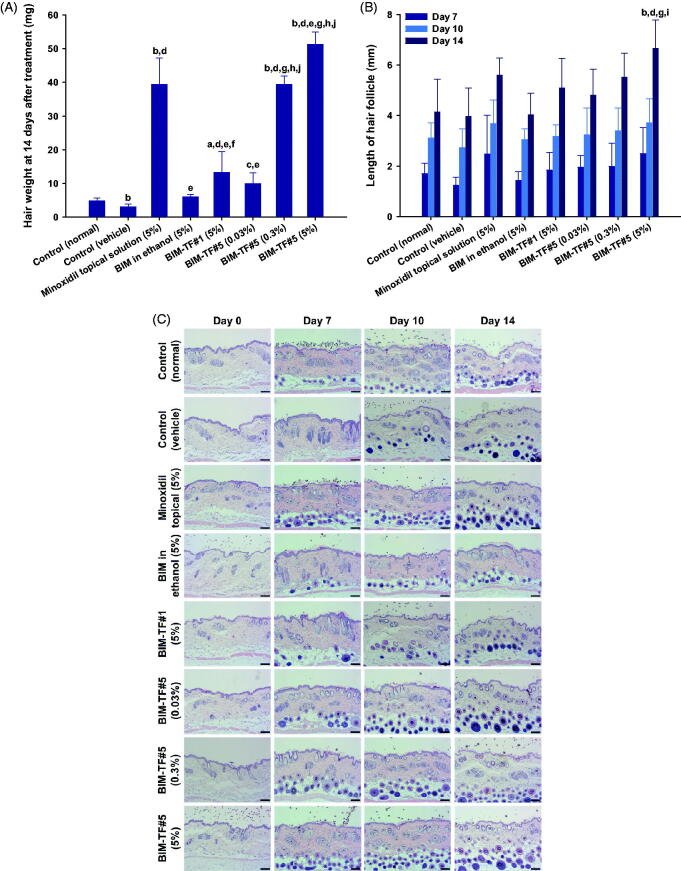
(A) Hair weights for each group at 14 days after treatment. (B) Length of hair follicles at 7, 10, and 14 days after treatment. ^a^*p*<.01, ^b^*p*<.001 compared to control (normal); ^c^*p*<.05, ^d^*p*<.001 compared to control (vehicle); ^e^*p*<.001 compared to minoxidil topical solution (5%); ^f^*p*<.05, ^g^*p*<.001 compared to BIM in ethanol (5%); ^h^*p*<.001 compared to BIM–TF#1 (5%); ^i^*p*<.05, ^j^*p*<.001 compared to BIM–TF#5 (0.03%). Values are means ± SDs (*n* = 8 for each group) (C) Representative light micrographs of sections from the androgenic alopecia skin, stained with hematoxylin and eosin (H&E) for observation of the number and diameter of hair follicles. Scale bar = 100 µm.

At the end of treatment, the newly grown hairs on the dorsal skin of all test groups were weighed (Figure 5(A)). The hair weight of the vehicle-treated group was less than that of the control (normal), suggesting delayed hair regrowth due to androgenic alopecia. The hair weight of the BIM–TF#5 (0.3%, w/w) treatment group was 1,198%, 549%, 197%, and 298% higher than for the control (vehicle), BIM in ethanol (5%, w/w), BIM–TF#1 (5%, w/w), and BIM–TF#5 (0.03%, w/w) groups, respectively (Figure 5(A)). Furthermore, at 14 days after treatment, androgenic hair regrowth efficacy for BIM–TF#5 (5%, w/w) was superior to the other treatment groups, including the control (normal) group; hair weight was 1593%, 418%, and 30.4% higher than for the control (vehicle), BIM–TF#5 (0.03%, w/w), and minoxidil topical solution (5%, w/v) groups, respectively (Figure 5(A)), suggesting that dense hair follicles formed after treatment with BIM–TF#5 (5%). Moreover, the lengths of hair follicles of the randomly plucked newly grown hairs in the dorsal skin were also measured at different time points. As shown in Figure 5(B), the hair follicles in the mice treated with BIM–TF#5 (0.03% [w/w], 0.3% [w/w], or 5% [w/w]) were longer than those of the control (vehicle) and control (normal) groups. In addition, seven days after treatment, follicles from the BIM–TF#5 (0.3%, w/w)- and BIM–TF#5 (5%, w/w)-treated mice were 1.60- and 2.02-fold longer than for the control (vehicle) group. At day 10, the follicle lengths of newly grown hairs were similar among all treatment groups. At 14 days after treatment, the length of hair follicles from mice treated with BIM–TF#5 (5%, w/w) was 6.66 ± 1.13 mm, which was 1.68-, 1.21-, and 1.19-fold longer than those from mice treated with vehicle, BIM–TF#5 (0.3%, w/w), and 5% (w/v) minoxidil topical solution, respectively.

To confirm the enhanced hair regrowth efficacy of BIM–TF#5 in androgenic alopecia mice, hair follicle density in the telogen area was histologically analyzed at various time points after treatment by staining with H&E (Figure 5(C)). After 14 days, the dorsal skin treated with vehicle BIM–TF#5 or BIM–TF#5 containing 0.03%, 0.3%, or 5% (w/w) BIM did not exhibit any signs of irritation or inflammation, such as epidermal thickening or infiltration of inflammatory cells. Topical application of BIM–TF#5 substantially increased the number of hair follicles compared to the normal and vehicle control, BIM in ethanol (5%, w/w), and BIM–TF#1 (5%, w/w) groups. Furthermore, the number and diameter of hair follicles on days 7 and 10 were significantly increased in the BIM–TF#5 (0.3%, w/w) and BIM–TF#5 (5%, w/w) treatment groups compared to all other groups, including the minoxidil topical solution (5%, w/v) group. Moreover, on day 14, BIM–TF#5 (0.03% [w/w], 0.3% [w/w], or 5% [w/w]) treatment was associated with more mature and thicker hair follicles in the deep subcutis compared to the other groups.

Taken together, the results indicate that the enhanced hair regrowth efficacy of BIM–TF#5 (5%, w/w) was due to immediate drug availability in deep dermal layers, leading to maximal direct activation of the human dermal papilla cells and keratinocytes; this elongated hair growth stage promoted the maintenance of hair follicles in the anagen phase compared to propylene glycol containing BIM–TF#1 (5%, w/w) and BIM dissolved in ethanol (5%, w/w). Further studies are required to optimize the treatment concentrations *in vivo*, and to determine the biological mechanisms underlying activation of the pathways promoting the telogen-to-anagen transition and extension of the anagen stage (which involves direct prostamide F2α receptor-mediated stimulation of dermal papilla cells within follicles). Additionally, hair regrowth efficacy in an androgenic female mouse model, as well as alopecia related to autoimmunity (e.g. alopecia areata), should be evaluated to demonstrate fully the effectiveness of the optimized BIM–TF#5 vehicle system against alopecia in both genders.

## 4. Conclusions

This study demonstrated that a ‘solvent mixture system’ for BIM including volatile and nonvolatile components significantly enhanced hair regrowth in denuded mice, by promoting immediate infiltration of the drug across full-thickness human skin. The optimal vehicle system (BIM–TF#5 containing 5% (w/w) BIM) showed 3.81-fold increased permeability and 6.29-fold increased dermal deposition of BIM relative to 5% (w/w) BIM in ethanol. In addition, 5 µM of BIM from BIM–TF#5 was associated with 12.4% and 8.42% greater proliferation of HaCaT and hDP cells compared to minoxidil (10 µM), and accelerated scratch-wound closure compared to other BIM–TFs. This suggests that BIM–TF#5 effectively promotes hair growth via biological activation of hair follicle cells. With BIM–TF#5 (5%), hair regrowth was greater at day 3; at day 14, skin treated with BIM–TF#5 (0.3% [w/w] or 5% [w/w]) and 5% (w/v) minoxidil topical solution, was completely covered by new hair. Moreover, newly grown hair weight in the treatment area was 12.0 mg higher for BIM–TF#5 (5%, w/w) than minoxidil (5%, w/v)-treated mice after 14 days of treatment, and hair follicles were 1.07 mm longer. In addition, hair follicle number and diameter at days 7 and 10 were greater for BIM–TF#5 (0.3% [w/w] or 5% [w/w] BIM) compared to the other BIM–TF groups and the minoxidil (5%, w/v) group. Notably, these results demonstrate that 0.3% (w/w) and 5% (w/w) BIM–TF#5 have therapeutic potential for alopecia.
